# Surgery of subacromial syndrome with application of plasma rich in growth factors

**DOI:** 10.4103/0973-6042.57932

**Published:** 2009

**Authors:** A Jiménez-Martin, J Angulo-Gutiérrez, J González-Herranz, J M Rodriguez-De La Cueva, J Lara-Bullón, R Vázquez-Garcia

**Affiliations:** Orthopaedic Surgery and Traumatology Service, University Hospital Nuestra Señora de Valme, Seville, Spain

**Keywords:** Acromioplasty, constant, cuff, DASH, PRGF, subacromial syndrome, UCLA

## Abstract

**Background:**

Our objective was to evaluate clinical recovery of patients with subacromial syndrome, after administering them plasma rich in growth factors (PRGF) by means of the Constant, University of California Los Angeles (UCLA) and Dissabilities of Arm, Shoulder and Hand (DASH) tests.

**Materials and Methods:**

Prospective cohort study involving two groups — group A, treated with PRGF (52 patients); and group B, without PRGF treatment (79 patients). We analyzed the clinical situation preoperatively (time 1), at 1 month (time 2) and after rehabilitation (time 3).

**Results:**

We considered 131 patients (71.2% were men, with median age of 53.7 years). Different approaches were used — traditional (62.5%), mini-open (22.5%) and arthroscopic (15%), without significant differences (P= .71). We observed improvement in the Constant test results at time 2 (59.8 ± 11.5 points in group A vs. 13.2 ± 7.1 points in group B; P < .05) and at time 3 (79.3 ± 11.6 points in group A vs. 59.7 ± 20.1 points in group B; P ¼ .05). We found improvement in the UCLA test results at time 2 (23.2 ± 5.8 points in group A vs. 4.72 ± 1.1 points in group B; P < .05) and at time 3 (32.1 ± 5.3 points in group A vs. 22.1 ± 7.35 points in group B; P < .05). We also observed improvement in the DASH test results at time 2 (45.2 ± 17.2 points in group A vs. 118.3 ± 7.6 points in group B, P < .05) and at time 3 (37.3 ± 12.6 points in group A vs. 69 ± 25.7 points in group B). Time of rehabilitation reduced significantly: 2.53 months in group A vs. 4.96 months in group B (*P* < .05). No significant differences were observed in surgical times: 88 minutes (group A) vs. 97 minutes (group B).

**Conclusion:**

In our experience, PRGF should be indicated in subacromial syndrome and cuff involvement, as shown by the improvement in our results in terms of better results of tests, reduction in rehabilitation time and no increase in operation time.

## INTRODUCTION

Subacromial syndrome is a very frequent pathology in our society, with a prevalence of 47% of population, from 5% to 80% in patients of over 80 years of age.[1]

Pathophysiology of the inflammatory process in subacromial syndrome has been studied by authors such as Kasperk[2] and Sakai.[3] However, there are no studies on biological repair of these lesions by direct application of plasma rich in growth factors.

## MATERIALS AND METHODS

Our objective was to evaluate the clinical effectiveness of PRGF in subacromial syndrome. We used numerical values of the validated Constant,[4] UCLA[5] and DASH[6] tests. We also measured influence of PRGF application on rehabilitation time (measured in months) and surgery time (measured in minutes). It was a comparative study of cohorts, between a prospective cohort (treated with PRGF); and a historical cohort, although recent, where PRGF was not applied. With the exception of PRGF addition, surgical techniques did not differ between the two cohorts. They were implemented according to the surgical protocols of the Orthopedic Surgery and Traumatology Service of our center. Our inclusion criteria were 1. adult patients (between 18 and 75 years of age) of both sexes; 2. history of painful shoulder, diagnosed with clinical history and physical examination; 3. confirmation of diagnosis with magnetic resonance imaging (MRI); and 4. signing in acceptance of the terms of the "informed consent" document. Our exclusion criteria were infectious pathologies, immunodepression or concurrent tumors. Other contraindications were as described by Sanchez[7]: alterations in platelet function, hemoglobin lower than 11 g/dL or hematocrit level lower than 34% and anticoagulants. Diabetes and steroids therapy were not considered contraindications.

Patients were divided into two groups:

Group A: Prospective cohort of 52 patients, from January 2006 to October 2007, with application of PRGF.
Group B: Historical cohort of 79 patients; they were operated from July 2004 to October 2007 at our center, with the same surgical techniques as used for group A, with the exception that they did not receive PRGF.

**Figure 1:**
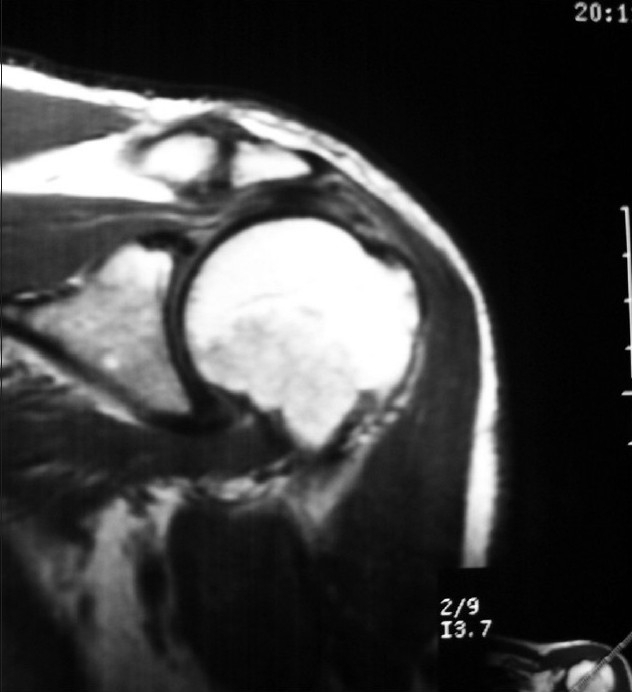
Tear of the rotator cuff (Observe the discontinuity in the fibers of the rotator cuff.)

Patients were evaluated with Constant and Murley,[4] UCLA[8] and DASH outcome measure tests[9] at 3 points in time: Time 1 was considered as the preoperative moment; time 2, at 1 month from surgery; and time 3 was at the end of rehabilitation. Performance of magnetic resonance imaging (MRI) preoperatively and postoperatively [Figure 1] and clinical evaluation and follow-up after several months were done to ascertain tendon improvement. Surgical methodology considered three different techniques: (a) traditional open surgery by Neer's approach; (b) mini-approach technique (mini-open), with or without arthroscopic support; (c) exclusively arthroscopic approach. It was considered that accomplishment of the three mentioned techniques and their distribution was homogeneous in the patients of both the groups (groups A and B). In group A, autologous plasma rich in growth factors was applied. Our main variable was the result of the Constant, UCLA and DASH tests. We also considered rehabilitation time and surgery time as variables, and we expressed them in units as months and minutes, respectively. Other variables such as pre-anesthetic risk, type of anesthesia, type of surgical procedure, complications and rehabilitation guidelines were also analyzed.

### Statistical methodology

The sample size was estimated from the results of an initial pilot sample, with a population variability of 25 units, considering bilateral character of test and an α error of 5% and a power 1-b of 80%. The CTM V1.1 program of Glaxo Smith Kline was used to estimate the minimum size of the group of patients to whom PRGF would be applied. Group A, with PRGF, included 52 patients; and group B, without PRGF, included 79 patients. The descriptive analysis used means and standard deviations or medians and percentiles. The inferential analysis used the Student t test for comparison of averages for independent samples; or in the case of non-normal distributions, the nonparametric Mann-Whitney U test. The relationships between the qualitative variables and two groups were estimated by means of the chi-square test or Fisher's exact test. Changes in the before-after answers (between the first and second moments of time analyzed, as well as between the second and third) due to the experimental intervention were analyzed by means of the nonparametric test of marginal homogeneity, considering that for two related ordinal variables it was an extension of the nonparametric test of McNemar with binary responses to the multinomial responses. Data analysis was done with the program SPSS 14.0 for Windows. Design and execution of the study were assessed by the Research Unit and were approved by the Committee of Ethics and Clinical Trials of our institution.

### Technical preparation

**Figure 2:**
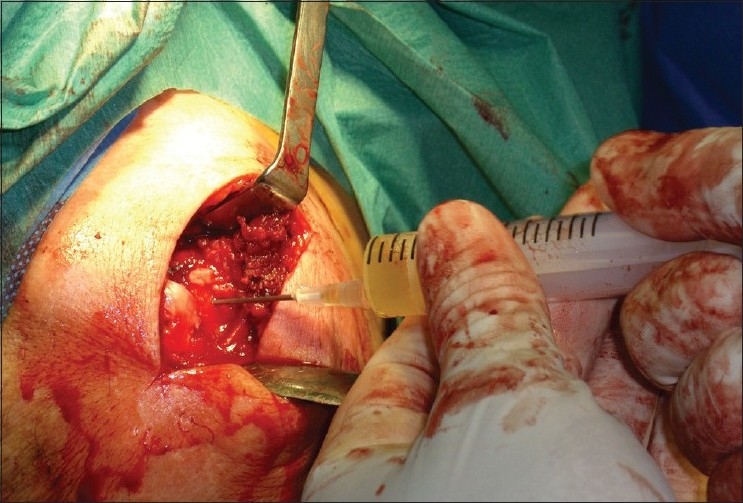
Application of PRGF over the suture

The usual acromioplasty was performed with repair of the rotator cuff, either in the mini-open or traditional manner, with or without arthroscopic support or in exclusively arthroscopic manner. Once this procedure was finalized, we injected the PRGF within the suture, and then a fibrin membrane cover was placed on this suture, in order to protect and facilitate the repair of the tissue [Figure 2]. To obtain the PRGF, 45 mL of venous blood was collected from the patient. It was stored in tubes with 3.8% trisodium citrate, and it was centrifuged at 1800 rpm for 8 minutes in order to separate different blood fractions (PRGF system II, BTI, Vitoria-Gasteiz,[10] Spain). This digital equipment works at 460G. Supernatants were then transferred to sterile tubes with sterile pipettes (sterilization with hydrogen peroxide), thus obtaining 3 different bottles containing plasma with different concentrations of growth factors. Next, the plasma was activated with addition of 50 mL of 10% calcium chloride for each milliliter of platelet-rich plasma fraction. Once the PRGF is activated, a clot is formed. We injected 9-10 mL of PRGF in all patients. They were separated on 3 fractions. One of them formed the clot in 7 minutes, due to its higher platelet concentration. The other ones were liquid. We applicated clot above rotator cuff and between it and shoulder. Afterwards, we injected platelet liquid fractions on suture.

## RESULTS

**Figure 3:**
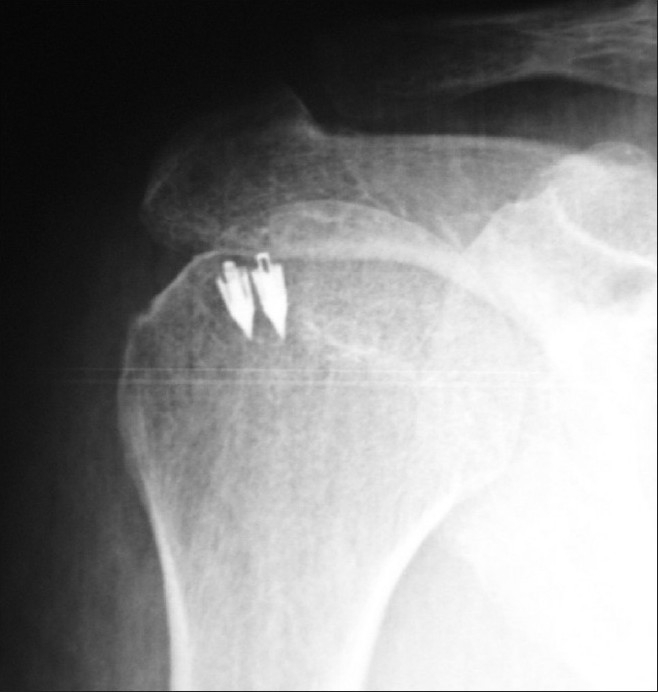
Positioning of anchors over the supraspinatus insertion or "footprint"

Group A included 52 patients; and group B, 79. Patients' clinical-epidemiological variables of sex, laterality, predisposing professions, concurrence with pathologies such as diabetes or arthrosis, as well as surgical techniques used in both groups, were similar, without statistically significant differences between them. Most patients were men in both groups (73.2% in group A and 74.1% in group B). The mean age of our patients was 53.7 ± 9.2 years (52 ± 10 years in group A vs. 51 ± 10 years in group B; P < .05). Positive Gerber, Yochum and Jobe's signs were presented in 69.2%, 95.2% and 85.30% of patients, respectively, with Neer and Yergason's positive tests in 69.2% and 16.1% of patients, respectively. Magnetic resonance imaging (MRI) showed impingement in 100% of patients and rotator cuff tears in 77.2% of global cases (100% in group A vs. 65.2% in group B, *P* = .0001). Traditional "open" procedure of acromioplasty and repair/ revision of rotator cuff were applied in 62.5% (69.5% in group A vs. 71.9% in group B), "mini-open" approach was applied in 22.5% (18% in group A vs. 22.2% in group B) and arthroscopy was done in 15% (12.5% in group A vs. 5.9% in group B) of the cases. There were no statistically significant differences between groups with regard to surgical procedures (*P* = .71). Globally, coracoacromial ligament was partially separated in 74%, osteophytes extirpation was done in 49.1% and Codman's area perforations were done in 53.4% of patients. The reinsertion technique was implemented with anchors [Figure 3]. We used anchors when rotator cuff insertion was damaged, especially if rotator cuff was retracted. We used lateral margin convergence[11] technique in fatty degeneration cases. We operated by means of acromioplasty alone on 37.5% of patients; and we carried out rotator cuff revision, but without suture. Rotator cuff repair with acromioplasty was realized on 62.5% of patients. We preferred open procedure to suture rotator cuff. Total ruptures represented 59.2% of cases. Longitudinal tears comprised a few cases, as the time period of evolution prior to surgery was several months, with high frequency of retraction.

Application of PRGF resulted in statistically significant differences in the results of Constant, UCLA and DASH tests, as shown in Tables 1–3. We evidenced pain reduction at 1 month of treatment in the group with PRGF. This reduction on visual analogue scale was higher in group A (from 10 to 5.2 points) than in group B (from 10 to 8.3 points), *P* = .06. Pain medication was the same in both groups, usually NSAIDs (especially ibuprofen).

**Table 1:** Results of Constant's test

Results of Constant's test	Median	Typical deviation
Group A (With PRGF)		
Results Constant Time 1	24.3	6.0
Results Constant Time 2	59.8	11.5
Results Constant Time 3	79.3	11.6
Group B (Without PRGF)		
Results Constant Time 1	11.8	5.1
Results Constant Time 2	13.2	7.1
Results Constant Time 3	59.7	20.1

Times: preoperative (time 1), at one month from the intervention (time 2) and at the end of rehabilitation (time 3)

**Table 2:** Results of the UCLA test

Results of the UCLA test	Median	Typical deviation
Group A (With PRGF)		
Results UCLA Time 1	6.70	2.3
Results UCLA Time 2	23.2	5.8
Results UCLA Time 3	32.1	5.3
Group B (Without PRGF)		
Results UCLA Time 1	2.6	2.5
Results UCLA Time 2	4.7	1.1
Results UCLA Time 3	22.1	7.3

Times: preoperative (time 1), at one month from the intervention (time 2) and at the end of rehabilitation (time 3)

**Table 3:** Results of the DASH test

Results of the DASH test	Time 1	Time 2	Time 3
Group A (With PRGF)			
Median	140.6	45.2	37.3
Typical deviation	5.2	17.2	12.6
Group B (Without PRGF)			
Median	124.2	118.3	69.0
Typical deviation	8.5	7.6	25.7

Times: preoperative (time 1), at one month from the intervention (time 2) and at the end of rehabilitation (time 3)

Application of PRGF resulted in a statistically significant reduction of rehabilitation time (2.5 ± 0.7 months in group A *vs*. 4.9 ± 1.8 months in group B; P < .05). Rehabilitation techniques used were kinesitherapy in 84.9%, mechanotherapy with pulleys in 74.4% and pendular exercises in 73.2%, without significant differences between both groups regarding the frequency of their application. There were no statistically significant differences between both groups in surgery time: (88 ± 21.3 minutes in group A *vs*. 97 ± 29.3 minutes in group B; *P* = .76).

In group A there were 2 cases of superficial infection that did not need antibiotherapy or re-interventions. There were no cases of anchor movement. Nevertheless, in group B, we found a case of adhesive capsulitis, a case of infection/ fistula that required surgical drainage and 2 cases of anchor movement. Persistent pain appeared in 27% of patients of group B *vs*. 7% of patients of group A (*P* = .024). Limitation of movement was observed in 22% of patients of group B *vs*. 5.2% of patients of group A (*P* = .018). Crepitation presented in 10% of patients of group B *vs*. 0% of patients of group A (*P* = .051). Hyper-abduction was persistently painful (27% in group B *vs*. 4.8% in group A; *P* = .0068). We did not find statistically significant differences between groups with regard to frequency of cheloidal scars; it was around 4% in both groups.

## DISCUSSION

Since 1972 when Charles Neer[1] introduced acromioplasty as a surgical technique, other alternative procedures have followed, such as MacFarland's lateral approach,[12] Cabot's mini-open technique[13] and Hata's procedure.[14] Nevertheless, Ellman[8] was one of the first authors who described arthroscopic acromioplasty and this technique became the first step of development of arthroscopic rotator cuff surgery. Arthroscopy has provided the best treatment of rotator cuff tears without the great limitation of tear length, due to Burkhart's margin convergence technique[11] or Meier's double-row technique.[15] In fact, Pagán Conesa[16] describes advantages of arthroscopic treatment in 80 patients with improvements in the results of Constant and UCLA tests in 80% of the cases. In subacromial syndrome, different authors have studied the influence of inflammatory mediators and diverse growth factors. Nevertheless, there are no studies in the literature regarding application of PRGF to rotator cuff.

It would be interesting to analyze the statistically significant improvements found in the results of Constant, UCLA and DASH clinical tests described in our “results” section [Tables 1–3]. We attribute these improvements to PRGF application, as both groups were comparable with regards to surgical approach or epidemiological data. It would also be interesting to note that surgery times between both groups did not differ significantly. This fact and improvements in results of tests justify the enthusiasm of our group for the application of PRGF in patients with rotator cuff involvement.

The interest in tendinous tissue repair is a result of the studies by Takahasih,[17] who identified how fibroblastic growth factor b (recombinant b-FGF) had a stimulator effect on cellular proliferation in the rotator cuff, proposing that b-FGF could be the best potential therapy in initial phases of rotator cuff treatment when cellular proliferation begins and collagen I synthesis has still not begun. On the other hand, Kobayashi[18] pointed out that in rotator cuff, b-FGF expression takes place between postoperative day 1 and day 56, with a peak around day 7, and it is essential for collagen III formation and probably for rotator cuff tears repair. Furthermore, application of the transforming growth factor beta1 (TGF-β1) causes an increase in the levels of smooth muscle actin (SMA). These cells, which contain SMA in the rotator cuff, could constitute a collagen-glycosaminoglycan matrix analogous to an extracellular matrix in vitro. SMA is a well-known determinant in the recovery of the cuff, with a more rapid recuperation from tears. Finally, there have been some unsuccessful attempts to repair the damaged cuff with substitutes such as those described by Dejardin,[19] which tried to repair rotator cuff tears in dogs using grafts of intestinal submucosa. Also Awad[20] has studied the application of collagen membranes with mesenchymal stem cells from bone marrow, reporting greater forces of resistance against maximum rupture stress, without mechanical or ultrastructural differences in tendons.

Application of these growth factors could accelerate the tendon repair process within the damaged rotator cuff, which is the hypothesis that forms the basis of this work; it is interesting, considering that patients of group A presented with a greater percentage of rotator cuff involvement and had earlier functional recovery at the end of this study. This idea would support PRGF application in cases of subacromial syndrome, as there were no statistically significant differences between surgical procedures.

In fact, we have observed a significant reduction in rehabilitation time (2.53 *vs*. 4.96 months). We attribute this improvement to the PRGF.[21] We consider that this preparation is able to improve the recovery of rotator cuff or even accelerate it.[22]

Since Gibble[23] described the first "fibrin glue," different preparations have followed,[24,25] like autologous growth factors (AGFs)[25] used in spinal fusions. However, in our experience, PRGF is currently the best option. It has some advantages. This procedure uses sodium citrate as an anticoagulant and calcium chloride as an activator. The calcium chloride contribution causes native thrombin formation, attempting to imitate physiological process of coagulation and release of growth factors, which would be very useful for tissue repair. This procedure avoids the presence of immunological reactions, as well as transmission of contagious diseases, associated with the use of thrombin of bovine origin.[26] Also, PRGF contains around 600,000 platelets/μL, which is the optimal concentration to achieve a high and correct therapeutic benefit.[27] Weibrich[28] recommends a platelet concentration of approximately one million platelets per milliliter. It does not contain leukocytes, with metalloproteases such as 8 and 9 (MMP-8, MMP-9). These metalloproteases release oxidative radicals which cause cellular destruction of healthy as well as damaged structures in surroundings.[27] The fact that PRGF does not contain leukocytes[29] (with pro-inflammatory cytokines, like IL1, 6 and 8) means a much smaller inflammatory response, unlike other concentrated platelet preparations. Finally, cases of tumor development have not been described with its application.[10,30]

Clinical utility and tendon surgery advantages of PRGF have been studied recently. For example, Eduardo Anitua and Mikel Sanchez[30] studied the possible effects on the repair of Achilles tendons in lambs, this tendon being previously cut and later repaired with or without PRGF. The results of this group demonstrated that the presence of platelets in fibrin matrix increased tendinous cells proliferation significantly, especially in cases of platelet-poor plasma as compared to platelet-rich plasma (47,362 ± 5,624 vs. 65,268 ± 8,221 cells/cm^2^, respectively; *P*= .0013). Additionally, the cultivated tendon cells synthesized collagen I and angiogenic factors such as VEGF and HGF. PRGF application caused an increase in ovoid cells density in Achilles tendons of lambs and an increase in vascularization, without inflammatory proliferation, opening the door to new biological alternatives of treatment for these tendons. The same group has studied application of PRGF on Achilles tendons of human beings[7] ; in particular, athletes who received PRGF recovered to a previous range of mobility in 7 ± 2 weeks as opposed to 11 ± 3 weeks for the recovery without PRGF (*P* = .042). Mikel Sanchez has also used it in sports medicine in articular cartilage avulsions of the knee in soccer players.[21]

About rotator cuff tears, more recently, several authors, like Gulotta[31] or Rotini,[32] have described utility of growth factors in rotator cuff surgery. These proteins would induce mitosis, extracellular matrix production, neovascularization, cell maturation, and differentiation. For example, Kovacevic[33] used a sheep infraspinatus repair model to evaluate the effect of osteoinductive growth factors and BMP-12 on tendon-to-bone healing. Magnetic resonance imaging and histology showed increased formation of new bone and fibrocartilage at the healing tendon attachment site in the treated animals, and biomechanical testing showed improved load-to-failure. Everts[34] has demonstrated that platelet-leukocyte gel (PLG) is useful in rotator cuff surgery. Recovery was faster and patients returned earlier to daily activities and also took less pain medication than control subjects (*P* < .001).

Rodeo[35,36] too supports these new concepts in rotator cuff surgery. He affirmed in 2007 that magnetic resonance imaging showed that the volume of newly formed bone (*P* < .05) and soft tissue (*P* < .05) in the tendon-bone gap was greater in growth factor–treated animals compared with collagen sponge alone–treated control group. Histologic analysis showed a fibrovascular tissue in the interface between tendon and bone, and repairs that were treated with the osteoinductive growth factors had significantly greater failure loads at 6 weeks and 12 weeks (*P* < .05). Our study supports these hypotheses.

One limitation of the present study is its design. The ideal design to ascertain the effects of PRGF would be a randomized clinical trial or, at least, a comparison between two prospective cohorts. However, as we were convinced of PRGF benefits, we thought it would be more ethical to choose a comparative study, comparing one prospective cohort (with PRGF) with one recent historical cohort (without PRGF). In order to avoid the usual shortcomings of this kind of designs, we ensured that there were no significant differences between the two cohorts, with regard to patients' characteristics or the surgical techniques used.

## CONCLUSIONS

The results of the present study clearly indicate that PRGF application with acromioplasty and rotator cuff repair should be indicated in the pathology of subacromial syndrome. In our experience, PRGF improves clinical results of the Constant, UCLA and DASH tests and shortens rehabilitation period, without increase in surgery time.
